# Toward risk adjustment in mental health in Israel: calculation of risk adjustment rates from large outpatient and inpatient databases

**DOI:** 10.1186/s13584-020-00373-6

**Published:** 2020-04-14

**Authors:** Yoav Kohn, Amir Shmueli

**Affiliations:** 1 the Donald Cohen Child and Adolescent Psychiatry Department, Jerusalem Mental Health Center, Eitanim Psychiatric Hospital, 90972 DN Tzefon Yehuda, Israel; 2 Hebrew University-Hadassah School of Medicine, Jerusalem, Israel; 3 Braun School of Public Health and Community Medicine of the Hebrew University and Hadassah, Jerusalem, Israel

**Keywords:** Mental health insurance reform, Risk adjustment, Capitation formula

## Abstract

**Background:**

In 2015, mental health services were added to the Israeli National Health Insurance package of services. As such, these services are financed by the budget which is allocated to the Health Plans according to a risk adjustment scheme. An inter-ministerial team suggested a formula by which the mental health budget should be allocated among the Health Plans. Our objective in this study was to develop alternative rates based on individual data, and to evaluate the ones suggested.

**Methods:**

The derivation of the new formula is based on our previous study of all psychiatric inpatients in Israel in the years 2012–2013 (*n* = 27,446), as well as outpatients in one psychiatric clinic in the same period (*n* = 6115). Based on Ministry of Health and clinic data we identified predictors of mental health services consumption. Age, gender, marital status and diagnosis were used as risk adjusters to calculate the capitation rates for outpatient care and inpatient care, respectively. All prices of services were obtained from the Ministry of Health tariffs. These rates were modified to include non-users using restricted models.

**Results:**

The mental health capitation scales are typically “humped” with regard to age. The rates for ambulatory care varied from a minimum 0.19 of the average consumption for males above the age of 85 to a maximum of 1.93 times the average for females between the ages of 45–54. For inpatient services the highest rate was 409.25 times the average for single, male patients with schizophrenia spectrum diagnoses, aged 45–54. The overall mental health scale ranges from 2.347 times the average for men aged 45–54, to 0.191 for women aged 85+. The modified scale for the entire post-reform package of benefits (including both mental health care and physical health care) is increasing with age to 4.094 times the average in men aged over 85. The scale is flatter than the pre-reform scale.

**Conclusions:**

The risk adjustment rates calculated for outpatient care are substantially different from the ones suggested by the inter-ministerial team. The inpatient rates are new, and indicate that for patients with schizophrenia, a separate risk-sharing arrangement might be desirable. Adopting the rates developed in this analysis would decrease the budget shares of Clalit and Leumit with their relatively older populations, and increase Maccabi and Meuhedet’s shares. Future research should develop a risk-adjustment scheme which covers directly both mental and physical care provided by the Israeli Health Plans, using their data.

## Introduction

Mental health services in Israel are included in the third addendum to the National Health Insurance Law (1994) [1], along with other services that until the law came into effect were provided by the state. Accordingly, most ambulatory and hospitalization services were provided in the past by government operated mental health clinics and hospitals, rather than by the Health Plans. This dichotomy of separation of psychiatric and other mental health services from the rest of health care is undesirable in the opinion of the majority of those concerned, primarily with regarding patients who suffer from psychiatric disorders. The inclusion of mental health in the basic health package provided by the Health Plans is essential for the following reasons:

1. Psychiatry is a medical branch in every respect. The separation between physical and mental health is rooted in relatively new Western philosophical doctrines that are not accepted any more [2]. The scientific discoveries of medicine in the past seventy years have proven that all mental activity, both normal and pathological, has a correlation with brain activity. Much of the treatment of psychiatry today is pharmacological. In many psychiatric patients, there are accompanying medical problems that require integrated medical treatment. Treatment under the same framework is expected to improve its quality and efficiency [3, 4].
2. The separation between psychiatric care and other medical treatments perpetuates the stigma that has afflicted people with mental health problems.
3. The provision of mental health services by the state was done in the past without a clear definition of the package of services to which the residents of Israel are entitled, and without an incentive for the state to take care of the proper level of provision of services. For example, most of the services were provided in old buildings and without quality control of the treatment given, while patients waited in line for very long periods of time.

Since the enactment of the National Health Insurance Law, several failed attempts were made to include mental health services as part of the health package of services that the Health Plans are obliged to provide (“the mental health insurance reform”). In 2003, a decision to do so was made by the government, following which negotiations were held between the Ministries of Health and Finance and the rest of the parties involved, and in 2008–2012 an effort was made to implement the change in the framework of the legislation [5]. These efforts encountered many difficulties, among others, the objection of professionals who feared that the quality of service provided to patients would be impaired, and who suggested that government owned clinics should remain in their current format (the “benevolent reform”) [6]. In the end, a regulation was issued by the Minister of Health to include mental health services in the health services package as of 1/7/15 [7].

Towards the inclusion of mental health in the health services package, the Ministry of Health and the Health Plans began the process of evaluating and establishing new services [8]. For the purpose of expansion, the services package budget was increased by NIS 1.261 billion for hospitalizations and NIS 0.776 billion for ambulatory services.

Along with the clinical and therapeutic issues arising from this change, the issue of the allocation of the (additional) budget to the Health Plans should be considered. A new risk-adjustment (capitation) formula which covers (both) physical and mental health care is needed. The inter-ministerial team which was established for this issue [9] recommended allocating the mental health hospitalization funds according to the historical and expected share of the Health Plans in national mental health hospitalization expenditures. These calculations take into account the current process of decrease in the relative share of the largest Health Plan, “Clalit”, and increase in the share of the smaller Plans over the years (Table 1). The budget for ambulatory services is added to the existing (physical health care only) budget, which is allocated among the Plans by adding to the current risk-adjustment formula a new expenditure category – ambulatory mental health care - based on age only. This was done taking into consideration the goal of increasing usage of ambulatory services by 2% in persons below the age of 24, and 4% in persons above 25. Table 2 shows the changes in the pre-reform capitation rates, i.e., the mean cost of care in the risk-adjustment age-gender group relative to the overall mean cost of care in the population, implied by adding ambulatory mental health care as a separate "expenditure category. Basically, the change transfers funds that go to the Health Plans for insurees in advanced age to insurees in middle age.

**Table 1:** The recommended allocation of mental health hospitalization budget by the inter-ministerial team ^9^

Year	**Clalit**	Leumit	Maccabi	**Meuhedet**
**2015**	64.4%	8.4%	18.1%	9.2%
**2016**	63.3%	8.6%	18.6%	9.5%
**2017**	62.2%	8.8%	19.1%	9.9%
**2018**	61.1%	9.1%	19.6%	10.2%
**2019**	60.0%	9.3%	20.1%	10.6%
**2020**	59.0%	9.6%	20.6%	10.9%
**2021**	57.9%	9.8%	21.1%	11.2%
**2022**	56.8%	10.0%	21.6%	11.6%
**2023**	55.7%	10.3%	22.1%	11.9%

**Table 2:** Recommendation of the inter-ministerial team regarding the change in the capitation rates implied by adding ambulatory mental health care ^9^

age group	Difference for mental health	
	Male	Female
**Up to 1**	−0.02	−0.01
**1–5**	0.00	0.00
**5–15**	0.01	0.01
**15–25**	0.01	0.01
**25–35**	0.02	0.01
**35–45**	0.01	0.01
**45–55**	0.00	0.00
**55–65**	−0.01	−0.01
**65–75**	−0.04	− 0.03
**75–85**	− 0.06	−0.05
**above 85**	−0.06	−0.05

The inter-ministerial team’s risk-adjustment scheme has several shortcomings, most of them originated from lack of data. First, while payment for ambulatory mental health care is integrated into the payment for care provided by the Health Plans, it is done using the methodology of “expenditure heads”, a methodology which is not the common one used elsewhere and which is subject to major criticism. The major drawback is the assumption of equal unit-costs across health plans [10]. Second, the only risk adjuster used for ambulatory mental health care is age. Diagnosis, marital status and sex, for example, are not included although they exercise significant effects on mental health services use. Third, the payment for inpatient mental health care is determined by the historical and predicted shares of the Plans in mental health hospitalization. This procedure presents no incentives towards any social goal. Furthermore, it is not clear why the predicted share – even if accurate – indicates the (predicted) cost of care, since the Health Plans can substitute outpatient care for inpatient care and physical health care for mental care. The recommended risk-adjustment scheme is thus clearly insufficient in preventing risk selection, skimping on quality and quantity of care and distortion of the competition among the Health Plans [11].

The international experience with risk adjustment in mental health services shows a weak-moderate prediction of psychiatric services consumption based on different a variety of demographic and clinical variables. The RAND study examined a subset of insured patients in California with high expenditures on mental health during the years 1991–1994 [12]. Gender, age, ethnic origin, marital status, entitlement to social services, psychiatric diagnoses, a functional index and past expenditure, together predicted up to 16% of the variance of expenditure on mental health. Another study from the Veterans Administrations in the US [13] found that gender, age, medical and psychiatric diagnoses, and alcohol and drug abuse, predicted up to 20% of the variance of the expenditure on mental health and 32% in general health expenses. In the Netherlands, a separate risk-adjustment scheme was in effect until last year, when a reform combined physical and mental health care.

The goal of this study is to propose a risk adjustment scheme for mental health based on expected cost of patients classified by age, gender, marital status and diagnosis, to derive overall post-reform risk adjustment rates based on age and gender, and to examine how a formula based on these rates would effect the allocation of the budget among the Health Plans. This would help correct the current situation in which the hospitalization portion of mental health is not divided capitally but by a linear formula. As we have only partial data, this study is aimed at providing a “proof of concept” that such a formula, more accurately calculated with full data, can be derived and should be adopted by policy makers.

## Methods

The risk-adjustment rates signify the mean cost of care in the risk-adjustment group (defined by the choice of the risk-adjusters) relative to the overall mean cost of care in the population. The derivation of the risk adjustment rates in this study consists of two steps: first, based on utilization data and the selected risk adjusters, the rates are calculated for the users, namely, those who have a non-zero cost of care. Second, in each group, the rates are recalculated taking into account the number of non-users, namely those with zero cost. Because of limited data on the prevalence of the use of mental health care in some groups defined by the risk adjusters above, the rates were calculated for a restricted model only.

In order to calculate the rates among the users, we used results from our previous study [14]. We collected data on all patients hospitalized in psychiatric wards in Israel in the years 2012–2013 (*n* = 27,446) available through the Israel Psychiatric Case Registry [15]. Days of stay were calculated only for the 2 years studied even if hospitalization started before or ended after this period. We computed the total expenditure on hospitalization for each patient.

Data on all patients receiving treatment in one psychiatric outpatient clinic in Kiryat HaYovel, Jerusalem (*n* = 6115) were also available. Although we can not argue that this clinic is an accurate representative of all mental health clinics in Israel, it does serve a heterogenous population of Jews and non-Jews, high and low socioeconomic groups and urban and rural populations residing in West Jerusalem and the vicinity. For these patients we were able to calculate expenditure on ambulatory mental health care. This was done by multiplying numbers of ambulatory visits by the respective Ministry of Health tariff. Regression models were used to predict expected expenditures. Three models were estimated: the “demographic model” (including age and gender as risk adjusters), the “socio-economic model” (where socio-economic variables were added), and a “clinical model”, where diagnoses were added [14].

Because of the limited data on use in the groups defined by the models above, for the calculation of the rates for ambulatory care, we used the “demographic model”. For inpatient care, a “restricted demographic-socioeconomic-clinical” model was constructed. This model includes gender and age groups as well as the two strongest predictors we identified in our analysis: whether the person is single or not and has a schizophrenia spectrum diagnosis (SSD) or not. SSD was the most prevalent diagnostic group in inpatients in our study (51% compared to 1.6–15% for other diagnostic groups). Also, much less is known about the prevalence of the other diagnostic groups in different age and gender groups and their relationship to being single, data that were needed for calculations of weights.

Data on the number of hospitalized patients in each age and gender group in the study period were taken from the Israel Psychiatric Case Registry. Data on the number of patients in public mental health clinics in Israel during the study period in each age – gender group were obtained from the Ministry of Health Statistical Report for 2012 [16]. Data on the number of individuals residing in Israel in the different age and gender groups were taken from the Statistical Report of the Central Bureau of Statistics for 2012 [17].

For SSD it was possible to estimate the size of the relevant subgroups in the general population, based on data from the literature: The prevalence of schizophrenia in the general population is 1%, the average age of onset is 10–25 for men and 25–35 for women [18]. The prevalence of marriage among schizophrenia patients is lower than the average in the population. Data on the prevalence of marriage among schizophrenia patients were found for various developing countries in the world. No data were found for developed countries. A proportion for 20% of married individuals among individuals diagnosed with schizophrenia was chosen according to data from Sao Paulo, Brazil, the population with the closest socioeconomic status to Israel among those reported [19]. These estimates of schizophrenia and single status in schizophrenia were used to assess the size of subgroups in the population for different age groups and genders. The number of hospitalized patients in each subgroup was taken from the Psychiatric Case Registry data for the study period.

The ambulatory and inpatient capitation rates were weighted by the share of each service in total mean mental health care cost in the population as found in the study (NIS 12 and NIS 166 respectively) and by their shares in the budget as determined by the inter-ministerial team (1.8 and 2.8% respectively) to create capitation rates for (total) mental health care. These latter rates were further combined with the pre-reform (physical health care) capitation rates using their shares in mean cost, NIS 178 for mental health (this study) and NIS 4554 for physical health (for the year 2014), and in the budget (4.6 and 95.4% respectively) to produce overall post-reform risk adjustment rates. Finally, the shares of the Health Plans in the post-reform budget – determined by their shares in total “standardized insurees” according to the age-gender composition of their insured population (data from the National Insurance Institute for November 2015) - were calculated for both the inter-ministerial team’s recommendations and our overall capitation rates.

## Results

### Risk adjustment rates for ambulatory care (clinic data)

Table 3 presents the resulting risk adjustment rates for ambulatory care using the “demographic model”. The rates are higher for ages 25–64, and for women. The expected cost on ambulatory mental health care among women aged 45–54, for example, is 1.9346 times the mean cost of that service in the population.

**Table 3:** Capitation rates for ambulatory mental health care based on clinic data (“Demographic Model”)

Age Group	Male	Female
**0–14**	0.4260	0.4492
**15–24**	0.8717	0.9201
**25–34**	0.9776	1.4003
**35–44**	1.2989	1.3691
**45–54**	1.2388	1.9346
**55–64**	1.4396	1.5335
**65–74**	0.6244	0.6839
**75–84**	0.3359	0.3954
**Over 85**	0.1920	0.2515

### Risk adjustment rates for hospitalization (inpatient data)

Tables 4-5 present the rates for mental health hospitalization using the “demographic” and the “restricted” models respectively. Table 4 shows that with regard to age, as was found for ambulatory care above, the scale has a hump in middle ages (as opposed to the physical health care rates which increase with age). Men tend to have higher cost of inpatient care than women.

**Table 4:** Capitation rates for mental health hospitalization based on hospital data (“Demographic Model”)

Age Group	Male	Female
**0–14**	0.1553	0.0710
**15–24**	1.2383	0.6029
**25–34**	2.0629	0.7822
**35–44**	2.1303	0.9272
**45–54**	2.4276	1.2784
**55–64**	2.1754	1.4210
**65–74**	1.2805	0.9770
**75–84**	0.6411	0.5984
**Over 85**	0.3703	0.1863

**Table 5:** Capitation rates for mental health hospitalization based on hospital data (“Restricted demographic-socioeconomic-clinical model”)

Age Group	SSD and single		SSD and not single		No SSD and single		No SSD and not single	
	Male	Female	Male	Female	Male	Female	Male	Female
**0–14**	317.85	120.86	0.00	0.00	0.12	0.06	0.00	0.00
**15–24**	57.34	17.41	162.01	47.79	0.49	0.37	0.91	0.35
**25–34**	104.62	69.60	90.90	32.44	1.02	0.58	0.31	0.18
**35–44**	261.11	86.53	81.50	43.67	2.44	0.84	0.40	0.25
**45–54**	409.25	117.75	97.26	68.10	3.36	0.98	0.65	0.42
**55–64**	359.40	156.92	81.17	75.96	4.01	1.24	0.75	0.50
**65–74**	167.27	103.14	33.29	34.33	2.16	1.07	0.54	0.45
**75–84**	141.86	86.07	26.62	26.62	1.75	0.84	0.39	0.31
**Over 85**	0	0	19.84	18.79	1.31	0.61	0.24	0.16

*SSD* schizophrenia spectrum diagnosis

The rates based on the “restricted” model are calculated for age, gender, being single and having a schizophrenia spectrum diagnosis, are presented in Table 5. Since this calculation is based on many assumptions and approximations, its results should be treated with caution. However, it can be seen that single individuals with schizophrenia spectrum diagnoses have hospitalization costs up to 300–400 times the average in the population in men and up to 120–160 in women. Men with schizophrenia spectrum diagnoses who are not single are 100–160 times more costly than the average in the general population. For women the increase is 75 fold. Being single without a diagnoses of schizophrenia spectrum disorders leads to an expenditure of up to 4 times the general average in men, with no significant increase in expenditure for women. Individuals who are not single and without a diagnosis of schizophrenia spectrum disorders spend far less than the average in both genders.

### Risk adjustment rates for mental health care

Table 6 presents the overall rates for mental health care based on the “demographic models”. As explained in the Methods section, the ambulatory scale in Table 3 (which is assumed to represent the national scale) and the inpatient scale (Table 4) are combined twice, one with weights based on costs, and second, with weights based on the budgets. The scales have similar shape as in Tables 3-4. The scales derived using weights based on cost are generally higher than those based on the budgets, since the cost-based weight of hospitalization is higher than the budget-based one.

**Table 6:** Capitation rates for total mental health care based on clinic and hospitalization data (“Demographic Model”)

Age Group	Services’ weights based on costs		Services’ weights based on budgets	
	Male	Female	Male	Female
**0–14**	0.174	0.096	0.261	0.219
**15–24**	1.214	0.624	1.095	0.727
**25–34**	1.990	0.824	1.638	1.024
**35–44**	2.074	0.957	1.805	1.100
**45–54**	2.347	1.323	1.962	1.535
**55–64**	2.126	1.429	1.887	1.465
**65–74**	1.236	0.957	1.024	0.862
**75–84**	0.621	0.585	0.522	0.519
**Over 85**	0.358	0.191	0.301	0.212

### Modified risk adjustment rates for the entire post-reform package of benefits

The modified post-reform overall rates are shown in Table 7. Since the cost-based and the budgets-based weights of mental health care in total health care are very close (4%), the resulting scales are close. They are monotonically increasing with age, and the men’s scale is higher in advanced age, and lower in younger ages – the contribution of the physical health care scale. In comparison with the pre-reform physical health care scale, the modified one is flatter – the rates are higher in middle ages, and lower in advanced ages.

**Table 7:** Capitation rates for total health care

Age Group	Services’ weights based on costs		Services’ weights based on budgets	
	Male	Female	Male	Female
**0–14**	0.651	0.545	0.651	0.547
**15–24**	0.402	0.447	0.403	0.453
**25–34**	0.479	0.743	0.476	0.753
**35–44**	0.636	0.796	0.636	0.804
**45–54**	1.051	1.156	1.044	1.168
**55–64**	1.812	1.699	1.804	1.699
**65–74**	3.078	2.567	3.052	2.549
**75–84**	4.008	3.294	3.974	3.267
**Over 85**	4.094	3.395	4.059	3.368

### The effect on the allocation of the budget to the health plans

Adding services to the package of benefits with their different risk-adjustment rates changes the pre-reform rates, and hence – the shares of the Health Plans in the total budget, according to their age-gender composition. Table 8 presents four allocations: the pre-reform one (December 2014), the post-reform actual one (implied by the inter-ministerial team’s recommendations, December 2015), and two allocations implied by our post-reform modified scales (Table 7).

**Table 8:** The shares of the Health Plans in the 2015 budget of the package of benefits (covering physical and mental health care) implied by the various sets of capitation rates (%)

	**Clalit**	Leumit	Maccabi	**Meuhedet**
**Pre reform (December 2014)**	55.68	8.40	11.92	24.00
**Post reform -The inter-ministerial team’s recommendation (December 2015)**	55.33	8.30	12.05	24.32
**Post-reform - This study (“Demographic model”) Weights based on costs (November 2015)**	54.84	8.28	12.17	24.72
**Post-reform - This study (“Demographic model”) Weights based on budgets (November 2015)**	54.81	8.28	12.18	24.73

The mental health care reform clearly increased the shares of Maccabi and Meuhedet, at the expense of Clalit and Leumit. The reason is that mental health care utilization is higher in middle age groups, which are more represented in Maccabi and Meuhedet. Our modified rates introduce a somewhat sharper decline in the shares of Clalit and Leumit and higher increases in Maccabi and Meuhedet’s shares than the actual ones. The differences are small, but notice that a 0.1% share means NIS 40 million.

## Discussion

Based on previously identified predictors of mental health expenditure we were able to calculate rates for the capitation formula by age and gender in ambulatory care. For inpatient expenses, the rates were calculated adjusting for gender, age, being single, and a diagnosis of schizophrenia spectrum disorders. The range of values ​​of the calculated rates illustrates how different the risk may be between different groups of insured individuals.

The rates we calculated are different from what was proposed by the inter-ministerial team that dealt with this subject. The team did not suggest rates for the hospitalization expenses, and as can be seen from our results these vary widely between different age and gender groups, and much more when a schizophrenia spectrum diagnosis and singlehood are added.

Failing to account for that heterogeneity might lead to risk selection, skimping on quality and quantity of care and distorts the competition among the Health Plans.

Two limitations of the study should be mentioned: The calculation of the rates for the entire population (including non-users) is based on “imported” parameters, which might not reflect the Israeli reality. Second, our ambulatory scale is based on one – not necessarily representative – clinic.

## Conclusions

The risk adjustment rates we calculated based on individual data are substantially different from the ones suggested by the inter-ministerial team that dealt with allocation funds for mental health in Israel. The inter-ministerial team chose to allocate the inpatient mental health funds according the share of the Health Plans in total mental hospitalization historical cost, which implicitly assumes that all mental health inpatients and days are similar. Since this assumption is clearly violated in practice, it creates incentives to risk selection and distortions.

While this study suffers from several limitations, it sheds some insights into the mental health risk adjustment scheme. In particular, it shows the need to consider diagnosis (especially SSD) as risk adjustment. It seems that the care provided to SSD patients should be compensated separately (e.g. as a “severe condition”), which was not done yet. At present, after four years of practice under the reformed system, Health Plans individual data should be used to calculate a modified post-reform risk adjustment rates.

## Data Availability

The data that support the findings of this study are available from the Israel Ministry of Health and the Jerusalem Mental Health Center, but restrictions apply to the availability of these data, which were used under license for the current study, and so are not publicly available. Data are however available from the authors upon reasonable request and with permission of the Israel Ministry of Health and the Jerusalem Mental Health Center.
